# Effectiveness of metaverse-based nursing education on operating room patient safety

**DOI:** 10.1371/journal.pone.0329650

**Published:** 2025-08-28

**Authors:** Jieun Shin, Nam-Yi Kim

**Affiliations:** 1 Department of Biomedical Informatics, College of Medicine, Konyang University, Daejeon, Republic of Korea; 2 Department of Nursing, Konyang University, Daejeon, Republic of Korea; Farhangian Teacher Education University: Farhangian University, IRAN, ISLAMIC REPUBLIC OF

## Abstract

**Purpose:**

It is speculated that practical education that utilizes the metaverse can reduce the drawbacks of limited practice environments for nursing students and achieve the educational goals of clinical practice to improve the competency and academic satisfaction of nursing students. Metaverse can represent the actual clinical experience and participation of learners. This study aimed to develop metaverse-based nursing education content on operating room patient safety and verify its effects on nursing students’ learning transfer motivation, self-regulated learning, self-efficacy, and patient safety nursing.

**Methods:**

This study had a nonequivalent control group pre- and post-experimental design. Nursing education content was developed based on the ASSURE model. The content consisted of surgical hand washing, sterile gown wearing, patient confirmation, surgical counting, specimen management, and high-risk drug management. The control group underwent clinical training in the operating room, and the experimental group underwent practical training in the metaverse operating room.

**Results:**

The control and experimental groups included 19 and 25 participants, respectively. On comparing the study variables after the intervention, the difference between the control and experimental groups was not statistically significant, and the average score of the experimental group was generally higher. In particular, patient safety nursing scores improved from 3.99 to 4.79 in the control group and from 4.05 to 4.80 in the experimental group. Both groups showed significant improvement from pre- to post-intervention, with similar trends in educational outcomes observed across the two groups.

**Conclusion:**

Both training methods demonstrated comparable educational outcomes. These findings suggest that metaverse-based education has the potential to be a viable alternative to traditional clinical training in operating room patient safety nursing.

## Introduction

The 4th industrial revolution and the COVID-19 pandemic have accelerated the transition to a new educational paradigm called information technology (IT)-based e-learning in nursing education [1]. New non-face-to-face educational programs, such as online lectures and mobile apps, are being developed [2], and various attempts to develop practical education programs to replace clinical practice for nursing students have continued [3]. Among these innovations, metaverse-based learning an immersive virtual environment has emerged as a promising trend in medical education [4]. The metaverse encompasses technologies such as augmented and virtual reality, which enable realistic simulation and interaction beyond spatial constraints [5,6]. With the limitations in improving practical nursing skills through conventional lecture-style education or simple observation during clinical practice [7], it is expected that metaverse, which can represent the actual clinical experience and active participation of learners, would significantly impact nursing education [8]. However, despite the growing interest in metaverse-based education, prior studies have largely focused on its feasibility and learner satisfaction, with limited emphasis on rigorous comparative effectiveness or skill-based outcomes [9]. Furthermore, there remains a lack of structured, validated programs specific to operating room (OR) nursing education. This gap underscores the need for more robust evidence and targeted interventions an area this study seeks to address.

Particularly, in clinical practice related to OR nursing, the possibility of opportunistic infections is very high owing to the weakened immunity of surgical patients, necessitating thorough infection control and limiting the clinical experience of nursing students [10]. In addition, the number of ORs is limited, such that some nursing students do not experience OR practice during clinical training. It is believed that practical education that utilizes the metaverse can reduce the pitfalls of the limited practice environment and achieve the educational goals of clinical practice for nursing students.

A significant portion of operating room (OR) nursing practice is devoted to ensuring patient safety. In this study, the term patient safety nursing refers to the knowledge, skills, and clinical practices that nurses employ to prevent harm and promote safety throughout all phases of perioperative care. This concept is strongly emphasized in Korean perioperative nursing research as a critical element of person-centered and safety-focused practice. It encompasses core competencies such as infection control, patient identification, instrument counting, and effective communication, all of which contribute to surgical safety [11].

OR nurses play a central role in managing surgical procedures efficiently and safely. Their responsibilities include maintaining accurate intraoperative records, promptly responding to clinical demands with professional expertise, verifying patient identity, and counting instruments to prevent retained surgical items [12]. Additionally, they are accountable for the proper handling of surgical specimens, instruments, and equipment, and for implementing rigorous infection control protocols to protect patient outcomes [13]. Accordingly, this study aimed to develop and evaluate a metaverse-based educational program that integrates the principles of patient safety nursing in the OR. The goal was to enable nursing students to understand, apply, and practice these essential competencies within an immersive and realistic virtual environment.

Studies on online clinical practice of nursing students have reported improvements in their problem-solving ability [6], learning satisfaction [14], and clinical confidence [15]. It is necessary to confirm the effect of self-regulated learning [16] in practical education by applying metaverse learning, considering that interactions between instructors, learners, and performance in VR are possible. Self-regulated learning means that learners become the subject of their learning activities, check their learning motivation and goals, become the subject of decision-making in the learning process, and manage learning resources [17]. In addition, it is necessary to examine learning transfer motivation and self-efficacy from various angles to enhance the understanding and ability of the knowledge and skills that are the basis of patient safety nursing practice and to continuously maintain and improve them, as well as the capacity for patient safety nursing in the OR.

## Materials and methods

### Research design

**Fig 1:**
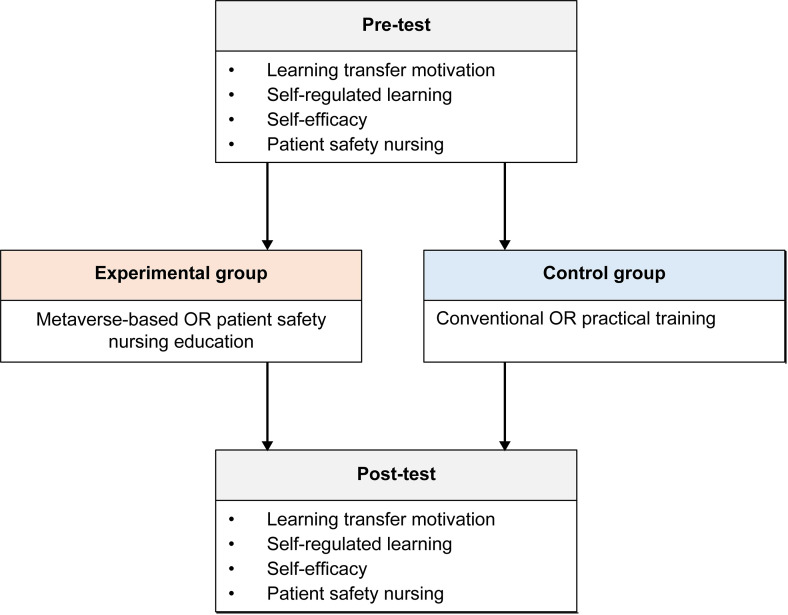
Flow chart of the study.

This study employed a nonequivalent control group pre- and post-experimental design to evaluate the effectiveness of a metaverse-based nursing education program focused on operating room (OR) patient safety. The study aimed to compare its impact on nursing students’ learning transfer motivation, self-regulated learning, self-efficacy, and competency in patient safety nursing with that of traditional clinical practice (Fig 1).

The educational intervention was developed based on the ASSURE instructional design model, which guided the systematic planning and delivery of the metaverse-based program. The ASSURE model consists of six essential steps: analyze learners, state objectives, select methods, media, and materials, utilize media and materials, require learner participation, and evaluate and revise [18].

1. Analyze learners: The educational needs of third-year nursing students were assessed through curriculum reviews, satisfaction surveys, and consultations with clinical instructors and industry partners. Key gaps were identified, especially in OR clinical experience due to limited access.
2. State objectives: Learning outcomes were clearly defined to include improvements in learning transfer motivation, self-efficacy, self-regulated learning, and patient safety nursing competencies.
3. Select methods, media, and materials: Based on learner needs and instructional goals, a metaverse platform was selected to overcome spatial and experiential constraints. Six core topics—surgical hand washing, sterile gowning, patient identification, surgical instrument counting, specimen management, and high-risk drug handling—were chosen in alignment with OR patient safety standards [13,19].
4. Utilize media and materials: Program content was developed collaboratively by nursing faculty and professional developers (Telos Co., Ltd). Each module included pre-learning orientation, instructor-led demonstration, learner avatar performance, quizzes, and Q&A sessions. Interactive scenarios and immediate feedback features were integrated to reinforce competency development.
5. Require learner participation: Students actively engaged in avatar-based practice within the virtual environment. They repeated tasks, responded to quizzes, and participated in voice-based Q&A sessions with instructors in real time.
6. Evaluate and revise: A pilot test was conducted with experts and students to assess content clarity, realism, and educational adequacy. Based on feedback, object visuals and procedural details were revised to enhance effectiveness.

This structured instructional approach ensured the pedagogical soundness of the virtual training and enabled a valid comparison with traditional OR clinical education. The overall structure of this quasi-experimental design is illustrated in Fig 1. Participants in both the experimental and control groups completed pre- and post-tests measuring learning transfer motivation, self-regulated learning, self-efficacy, and patient safety nursing competency. The experimental group received metaverse-based OR patient safety nursing education, while the control group underwent conventional OR practical training. This parallel structure allowed for direct comparison of the intervention’s effects, enhancing the internal validity of the study.

The specific objectives of this study were as follows:

- Primary objective: To compare post-intervention outcomes between the metaverse-based group and the conventional OR practice group.
- Secondary objective: To compare the change in outcomes from pre- to post-intervention between the two groups, as well as within each group.

This study aimed to develop metaverse-based OR patient safety education content and analyze its educational effectiveness. Based on a superiority framework, the study tested the following hypotheses:

- Hypothesis 1. Learning transfer motivation in the metaverse-based OR practice group will be higher than in the conventional OR practice group.
- Hypothesis 2. Self-regulated learning in the metaverse-based OR practice group will be higher than in the conventional OR practice group.
- Hypothesis 3. Self-efficacy in the metaverse-based OR practice group will be higher than in the conventional OR practice group.
- Hypothesis 4. Patient safety nursing in the metaverse-based OR practice group will be higher than in the conventional OR practice group.

### Participants and data collection

Experimental and control groups were recruited through the online community bulletin boards of two universities for nursing students in June 2024 (https://everytime.kr/). The control group was recruited from the nursing department of a university hospital where clinical practice in the OR was possible, and the experimental group was recruited from the nursing department of a university where clinical practice in the OR was not possible because of difficulties in securing a place for clinical practice in the OR. To prevent diffusion of the intervention effect between the control and experimental groups, universities that were distant from each other were selected. The selected individuals were 1) those enrolled in the third year of nursing, 2) those without clinical practice experience in the OR, and 3) those who agreed to participate in the study. The excluded subjects were 1) those in classes other than the third-year class of the nursing department, 2) those with clinical practice experience in the OR, and 3) those who refused to participate.

The number of participants was calculated using the G-power 3.1 program based on a previous study with an effect size of 0.8, power of 0.80, and significance level of 0.05 (two-tail), and the minimum sample size required was 26 participants per group [20]. Considering a dropout rate of 10%, 56 nursing students were recruited, including 28 nursing students each in the experimental and control groups [1]. The pre-intervention survey was conducted from July 8 to July 12, 2024. The intervention in the experimental group was conducted for 5 days from August 5 to August 9, 2024, and the post-learning survey was conducted 1 week later from August 19–23. The post-learning survey of the control group was conducted from September 23 to September 27, 1 week after the end of the clinical training.

In the control group, three participants were excluded due to missing responses in the pre-learning survey, four participants were excluded due to missing responses in the post-test survey, and two participants were excluded due to a leave of absence during clinical practice. In the experimental group, one participant were excluded because of missing responses in the post-learning survey, and two participants were excluded because of sudden connection problems during the training. The final numbers of participants were 25 and 19 in the experimental and control groups, respectively.

### Instruments

#### General characteristics of the study participants.

Demographic characteristics consisted of three items: age, sex, and academic performance in the previous semester.

#### Learning transfer motivation.

This study used an instrument developed by Ayres [21] and translated by Park, et al. [22]. The instrument comprises 10 items rated on a 7-point scale, with 7 signifying ‘very much so’ and 1 signifying ‘not at all so’ and higher scores indicating higher motivation for learning. The reliability of the instrument at the time of development (Cronbach’s α) was 0.80, and the reliability of the translated instrument was 0.94 [22]. The reliability in this study was 0.94 before and 0.94 after education.

#### Self-efficacy.

Among the learning transfer tools developed by Machin [23], the self-efficacy assessment tool was used, which consists of 12 items that are applied before education and 12 items that are applied after education. Each item in this tool is rated on a 7-point scale from ‘not at all (1 point) to ‘very much (7 points), with higher scores indicating higher self-efficacy. The reliability of the tool in the study by Machin [23] was 0.82, and in this study, it was 0.94 before and 0.97 after education.

#### Self-regulated learning.

Cho, et al. [24] developed and translated the self-regulated learning tool used in this study. This tool consists of 30 items that measure self-regulated learning in terms of interactions: 11 items on the student-learning content interaction aspect, 9 items on the student-professor interaction aspect, and 10 items on the student-student interaction aspect. This tool was rated on a 7-point scale, with higher scores indicating higher levels of self-regulated learning. The reliability of this tool in the study by Cho, et al. [24] was 0.94, and in this study, it was 0.96 before and 0.97 after education.

#### Operating room patient safety nursing.

OR patient safety nursing competency was revised and supplemented based on an instrument developed by Shin, et al. [25]. Items that are difficult for nursing students to perform (coordinating surgical schedules, preparing the OR for patients with infections, handling instruments, and checking for medical gases) were excluded from the study. As the items of the nursing education program developed in this study included content on wearing protective equipment, items related to wearing a surgical gown and sterile gloves and removing them after surgery were added. To examine whether each item of the revised and supplemented tool was related to the characteristics it was intended to measure, content validity was verified by two nursing professors in charge of OR nursing and practice, two nurses with more than 10 years of clinical experience who were working in the OR, and two managers of the patient safety department of a university hospital. The content validity index of the program was 0.8 or higher for all items; thus, it was considered appropriate.

The tool used in this study, which was composed of six sub-domains, comprised 38 items: infection control (8 items), patient identification (6 items), specimen management (5 items), surgical instrument count (8 items), high-risk drug management (4 items), and injury prevention (7 items). Each item was measured on a 5-point Likert scale ranging from 1 (not at all) to 5 (very much) according to the individual’s perception, with higher scores indicating higher competency in ensuring patient safety. The reliability of the measurement tool in the study by Shin, et al. [25] was Cronbach’s α = 0.91 and that in the present study was 0.95 before and 0.94 after education.

### Ethical considerations

This study was conducted after obtaining approval from the institutional review board of Kyungyang University for the protection of research participants (IRB No. KYU 2024-02-001-001). The recruitment notice and survey link were posted on the bulletin board of the online community of the college students to ensure autonomy of participation. Prior to the online survey, the study’s purpose and method, subject selection criteria, and other important details were explained to the subjects, and consent regarding the use of their personal information was obtained. Participants who checked “agree” before the survey could proceed to the next step. This study collected data online; thus, the IRB approved an exemption from written consent. The collected data would not be used for purposes other than the current research, and the participants were informed that they could quit the study at any time without penalties for the decision. The collected data will be stored for 3 years in a password-protected file on the personal computer of the principal investigator, which cannot be accessed by other individuals, and will be destroyed thereafter in a way that it cannot be restored.

### Mediation application and data collection

#### 1) Pre-intervention survey.

Pre-intervention surveys of the control and experimental groups were conducted simultaneously in July 2024. After the participants read an explanation of the research participation online and provided their consent, they proceeded to the survey stage. Participants were allowed to respond freely to a questionnaire on their general characteristics, learning transfer motivation, self-efficacy, self-regulated learning, and competency in OR patient safety nursing.

#### 2) Intervention.

The experimental group underwent the intervention, that is, the metaverse-based nursing education content on OR patient safety that we developed, in August 2024. OR patient safety nursing comprises six topics: surgical handwashing, sterile gown wearing, patient confirmation, surgical instrument counting, specimen management, and management of high-risk drugs. The metaverse platform allowed instructors to share program files so that students could participate; students could participate in the metaverse OR session on their personal computers. On the first day before the teaching, the participants logged into the metaverse OR and practiced by freely adjusting the avatar’s movements. Orientation was conducted on the OR structure, location of items, areas, educational topics, and schedule. Nursing education on OR patient safety was conducted from the second to the fourth day, and the topics were taught twice a day for 3 days. On the 5th day, we briefly reviewed patient safety nursing in the OR and held a Q&A section about other OR practice-related content (such as the names and uses of surgical instruments and the types of surgical sutures and needles) in the metaverse OR. The questions were organized in such a way that images of the surgical items are displayed when the names of the items are clicked. Each topic was taught for 50 minutes in the order: ‘pre-learning in the metaverse OR (15 minutes) – instructor demonstration (10 minutes) – learner performance (15 minutes) – quiz and review (5 minutes) – Q&A (5 minutes).’

**Fig 2:**
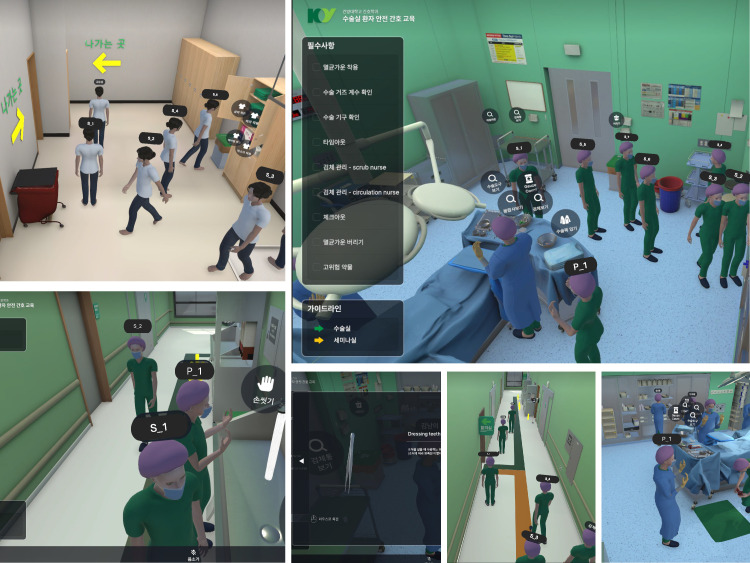
Metaverse operating room practice.

- **Pre-learning:** Participants logged into the metaverse OR, put on surgical gowns, caps, masks, and OR shoes in the changing room, and moved through the OR hallway (semi-contaminated area) to the seminar room. In the seminar room, the instructor briefly explained the topic, learning outcomes, prior knowledge, preparation materials, and procedures for each module.
- **Instructor demonstration:** After completing the pre-learning, the instructor and participants moved to a location in the OR that matched each topic. They learned by watching instructor demonstrations and related videos.
- **Learner performance:** Based on what the students learned from the instructor’s test and related videos, they moved the avatar to perform patient safety nursing in the OR. If any task was performed incorrectly (e.g., touching sterile surgical supplies without wearing a sterile gown), an alarm message was triggered to inform the students of the incorrect part of the performance. The learners’ performance allowed them to relearn freely within the given time (Fig 2).
- **Quiz and Review:** After completing the activities for each topic, the students gathered in the seminar room to take a quiz and review parts of the performance that were difficult or incorrect.
- **Q&A:** As the instructor and learners wore microphones during the metaverse-based OR training, they could freely ask questions on each topic related to patient safety nursing.

The control group comprised learners who underwent clinical training in the OR in September 2024. The clinical training lasted for 5 days and included surgical hand washing, sterile gown wearing, patient confirmation, surgical instrument counting, specimen management, and high-risk drug management in OR patient safety nursing. The control group received training similar to that of the experimental group, and the training was conducted in the following order: pre-learning (orientation) – instructor demonstration – learner performance – quiz and review – Q&A. However, unlike the experimental group, the control group received practical training in an actual operating room and not in the metaverse.

#### 3) Post-learning survey.

The post-learning survey of the experimental group was conducted 1 week after the end of the metaverse-based nursing education and that of the control group was conducted 1 week after the end of the clinical practice in September 2024. The control and experimental groups underwent the same survey on learning transfer motivation, self-efficacy, self-regulated learning, and nursing competency in OR patient safety as in the previous survey. The time required to complete the survey was 15–20 minutes.

### Data analysis

The collected data were analyzed using IBM SPSS Statistic 25.0 (IBM Corp., Armonk, NY, USA), and a two-sided test was applied at a significance level (α) of 0.05.

1) The general characteristics of the experimental and control groups and the characteristics of the research variables were calculated using descriptive statistics, such as numbers and percentages, means, and standard deviations.
2) The normality of the research variables was analyzed using the Kolmogorov-Smirnov test.
3) The homogeneity of the experimental and control groups was analyzed using an independent t-test.
4) Differences in pre- and post-learning transfer motivation, self-regulated learning, self-efficacy, and OR patient safety nursing between the experimental and control groups were analyzed using a paired t-test.
5) Differences in learning transfer motivation, self-regulated learning, self-efficacy, and OR patient safety nursing between the experimental and control groups were analyzed using an independent t-test. For the self-efficacy variable, which showed a significant baseline difference between groups, an analysis of covariance (ANCOVA) was performed using the pre-intervention score as a covariate to adjust for this initial difference.

Additionally, no statistical correction was applied for multiple testing; this may increase the risk of Type I error, so the results were interpreted with caution..

## Results

### Demographic characteristics of the study participants

Male participants comprised 21.1% of those in the control group and 4% of those in the experimental group; however, the difference was not statistically significant. The average age of the control group was 22 years and that of the experimental group was 20.96 years, with no significant difference. The average grades in the control and experimental groups were 4.16 and 3.72, respectively. The experimental group had a slightly lower score; however, the difference was not significant (Table 1).

**Table 1:** Comparison of Demographic Characteristics Across Groups.

	Control group	Experimental group	p
students (n)	19	25	
Sex (male)	4(21.1%)	1(4%)	.149^1^
Age	22 ± 2.29	20.96 ± 1.06	.078^2^
Past semester grades (out of 4.5)	4.16 ± 0.69	3.72 ± 0.84	.072^2^

^1:^ Fisher’s exact test.

^2:^ independent t-test.

### Normality test and homogeneity evaluation

Normality tests revealed that the study variables were normally distributed. The homogeneity test results for the two groups showed that the control group had an average of 5.53, and the experimental group had an average of 6.14 in self-efficacy, showing a significant difference. However, there were no differences between the control and experimental groups in terms of learning transfer motivation, self-regulated learning, or patient safety nursing competency (Table 2).

**Table 2:** Group Homogeneity Assessment.

		Control group	Experimental group	t	p
Learning transfer motivation		3.75 ± 0.93	4.09 ± 0.57	−1.406	.171
Self-efficacy		5.53 ± 0.89	6.14 ± 0.71	−2.520	.016
Self-regulated learning	Total	5.46 ± 1.13	5.59 ± 0.9	−0.429	0.67
	Student-learning content	5.47 ± 1.18	5.54 ± 1	−0.210	.834
	Student-professor	5.26 ± 1.32	5.3 ± 1.32	−0.112	.912
	Student-student	5.64 ± 1.16	5.92 ± 0.89	−0.901	.373
Patient safety nursing	Total	3.99 ± 0.59	4.05 ± 0.64	−0.330	.743
	Infection control	3.99 ± 0.71	4.30 ± 0.69	−1.474	.148
	Specimen management	3.52 ± 1.00	3.50 ± 0.83	0.043	.966
	Patient identification	4.41 ± 0.58	4.39 ± 0.56	0.148	.883
	High-risk drug management	4.28 ± 0.56	4.09 ± 0.98	0.742	.462
	Surgical item count	3.68 ± 0.93	3.78 ± 0.88	−0.331	.742
	Fall & bedsore prevention	4.14 ± 0.74	4.15 ± 0.64	−0.063	.950

### Analysis of intervention effects

#### 1) Before and after intervention group comparisons.

The results of comparisons before and after the intervention in each group are shown in Table 3. The mean values of the study variables generally increased in both groups, except for learning transfer motivation in the experimental group, which showed a slight decrease (from 4.09 to 4.08); however, this difference was not statistically significant.

**Table 3:** Pre- and Post-Learning Comparison by Group.

			Pre	Post	Differences	t	p
Control group	Learning transfer motivation		3.75 ± 0.93	3.99 ± 0.90	0.24 ± 0.47	2.237	.038
	Self-efficacy		5.53 ± 0.89	5.83 ± 1.03	0.30 ± 0.67	1.966	.065
	Self-regulated learning	Total	5.46 ± 1.13	5.68 ± 1.06	0.18 ± 0.77	1.002	.330
		Student-learning content	5.47 ± 1.18	5.65 ± 1.26	0.23 ± 1.01	1.005	.328
		Student-professor	5.26 ± 1.32	5.49 ± 1.23	0.25 ± 0.70	1.569	.134
		Student-student	5.64 ± 1.16	5.89 ± 0.91	0.80 ± 0.58	5.994	.000
	Patient safety nursing	Total	3.99 ± 0.59	4.79 ± 0.25	0.80 ± 0.58	5.994	.000
		Infection control	3.99 ± 0.71	4.89 ± 0.17	0.91 ± 0.64	6.211	.000
		Specimen management	3.52 ± 1.00	4.32 ± 0.71	0.80 ± 1.03	3.376	.003
		Patient identification	4.41 ± 0.58	4.91 ± 0.18	0.50 ± 0.52	4.182	.001
		High-risk drug management	4.28 ± 0.56	4.82 ± 0.27	0.54 ± 0.62	3.796	.001
		Surgical item count	3.68 ± 0.93	4.8 ± 0.33	1.11 ± 1.00	4.864	.000
		Fall & bedsore prevention	4.14 ± 0.74	4.87 ± 0.24	0.74 ± 0.69	4.651	.000
Experimental group	Learning transfer motivation		4.09 ± 0.57	4.08 ± 0.52	0.01 ± 0.51	0.078	.938
	Self-efficacy		6.14 ± 0.71	6.33 ± 0.78	0.19 ± 0.78	1.234	.229
	Self-regulated learning	Total	5.59 ± 0.90	5.79 ± 0.85	0.34 ± 0.58	2.969	.007
		Student-Learning Content	5.54 ± 1.00	5.88 ± 0.90	0.07 ± 1.03	0.344	.734
		Student-Professor	5.30 ± 1.32	5.37 ± 1.22	0.15 ± 0.66	1.150	.261
		Student-Student	5.92 ± 0.89	6.07 ± 0.79	0.75 ± 0.64	5.894	.000
	Patient safety nursing	Total	4.05 ± 0.64	4.80 ± 0.29	0.75 ± 0.64	3.383	.003
		Infection control	4.30 ± 0.69	4.78 ± 0.33	0.48 ± 0.69	3.445	.002
		Specimen management	3.50 ± 0.83	4.65 ± 0.45	1.14 ± 0.85	6.702	.000
		Patient identification	4.39 ± 0.56	4.82 ± 0.36	0.43 ± 0.59	3.653	.001
		High-risk drug management	4.09 ± 0.98	4.86 ± 0.29	0.77 ± 1.00	3.841	.001
		Surgical item count	3.78 ± 0.88	4.82 ± 0.36	1.05 ± 0.87	5.977	.000
		Fall & bedsore prevention	4.15 ± 0.64	4.87 ± 0.26	0.72 ± 0.64	5.629	.000

In the control group, there were significant differences in learning transfer motivation, overall patient safety nursing, and all sub-factors, and the student-student interaction area, a sub-factor of self-regulated learning. Learning transfer motivation increased by 0.24 points from an average of 3.75 to an average of 3.99 after the intervention, and student-student interaction increased by 0.25 points from an average of 5.64 to an average of 5.89. The overall competency in patient safety nursing increased by 0.80 points from an average of 3.99 to an average of 4.79. The sub-factors of patient safety nursing competency increased by 0.91 points for infection control, 0.80 points for specimen management, 0.50 points for patient confirmation, 0.54 points for high-risk drug management, 1.11 points for surgical item count confirmation, and 0.74 points for fall and pressure ulcer prevention.

In the experimental group, there were statistically significant differences in the overall self-regulated learning and student-student interaction domains and in the overall patient safety nursing competency and its sub-factors. Self-regulated learning increased by 0.20 points from an average of 5.59 to 5.79 after the intervention, and the student-student interaction domain, a sub-factor, increased by 0.75 points. Overall patient safety nursing competency increased by 0.75 points from an average of 4.05 to an average of 4.80 after the intervention. The sub-factors of patient safety nursing competency showed increases of 0.48 points for infection control, 1.14 points for specimen management, 0.43 points for patient confirmation, 0.77 points for high-risk drug management, 1.05 points for surgical item count confirmation, and 0.72 points for fall and bedsore prevention.

#### 2) Comparison after intervention.

Post-learning differences between the control and experimental groups were not statistically significant; however, the averages in the experimental group were generally higher than those in the control group. Specifically learning transfer motivation was higher in the experimental group (4.08) than in the control group (3.99), and self-efficacy was also higher in the experimental group (6.33) than in the control group (5.83). In self-regulated learning, the control group had an average of 5.68, and the experimental group had an average of 5.79.

In the subareas of self-regulated learning, except for the teaching area, the experimental group showed higher scores in all class and student areas. Patient safety competencies were similar at 4.79 and 4.80, respectively, in both groups. The experimental group showed higher scores in all sub-areas of patient safety competency, except for infection control and patient management (Table 4).

**Table 4:** Post-Learning Comparisons between the Groups.

		Control group	Experimental group	t	p
Learning transfer motivation		3.99 ± 0.9	4.08 ± 0.52	−0.385	.703
Self-efficacy^1^		5.83 ± 1.03	6.33 ± 0.78	0.098	.756
Self-regulated learning	Total	5.68 ± 1.06	5.79 ± 0.85	−0.381	.705
	Student-learning content	5.65 ± 1.26	5.88 ± 0.9	−0.689	.496
	Student-professor	5.49 ± 1.23	5.37 ± 1.22	0.316	.753
	Student-student	5.89 ± 0.91	6.07 ± 0.79	−0.693	.492
Patient safety nursing	Total	4.79 ± 0.25	4.8 ± 0.29	−0.155	.878
	Infection control	4.89 ± 0.17	4.78 ± 0.33	1.545	.131
	Specimen management	4.32 ± 0.71	4.65 ± 0.45	−1.790	.084
	Patient identification	4.91 ± 0.18	4.82 ± 0.36	1.122	.269
	High-risk drug management	4.82 ± 0.27	4.86 ± 0.29	−0.513	.611
	Surgical item count	4.80 ± 0.33	4.82 ± 0.36	−0.224	.824
	Fall & bedsore prevention	4.87 ± 0.24	4.87 ± 0.26	0.047	.963

^1:^ F (ANCOVA test).

## Discussion

This study developed metaverse-based educational content on OR patient safety nursing based on the ASSURE model and applied it to nursing students to evaluate its effectiveness. The main significance of this study is that it confirmed that the learning outcomes of metaverse-based OR practice were similar to those of actual clinical practice. Both the control and experimental groups showed improved competencies in OR patient safety nursing, with no differences observed between the groups. Although the outcomes were similar, this finding is consistent with several studies reporting that metaverse-based simulation can serve as a viable alternative to in-person clinical experience. For example, De Gagne et al. found that metaverse-integrated nursing education enhanced students’ confidence and clinical decision-making [19], while Yang and Kang demonstrated comparable skill acquisition between virtual and in-person psychiatric nursing training [1].

There was no statistically significant difference in learning transfer motivation between the control and experimental groups after the intervention, although the average score of the experimental group was slightly higher. In the control group, significant differences were observed between pre- and post-learning tests across all variables. In the experimental group, statistically significant improvements were found in total self-regulated learning and total patient safety nursing. The pre-learning test average of learning transfer motivation was 3.75 points for the control group and 4.09 points for the experimental group, showing no difference in homogeneity verification between the groups. However, it is slightly higher than that in a previous study [26] that conducted psychiatric nursing practice online. Since the participants enrolled in this study voluntarily during the university vacation period, there is a high possibility that learners who actively participated in learning were included; therefore, there may have been a selection error. Learning transfer motivation, the ultimate goal of practical training, is the confidence that learners can apply the knowledge and skills they have acquired through education in clinical situations [26]. Learning transfer motivation is influenced by organizational learning culture [27]. The control group is believed to have shown differences in learning transfer motivation between the pre- and post-learning tests because of the effect of their nursing experience in actual situations during clinical practice and while observing or participating in problem-solving in an organized manner. In contrast, the metaverse OR is designed to perform nursing in a limited environment (several organization members, patients, and limited space), and it is believed that there would not have been differences in patient safety because patient safety is hardly influenced by organizational learning culture. Technologies that can design diverse environments and provide learners with collaborative and kinesthetic communication in metaverse-based education should be further developed [28]. However, since there was no difference in learning transfer motivation between the control and experimental groups, it seems plausible that actual clinical practice and metaverse-based learning showed similar results.

There were no significant differences in self-efficacy between the control and experimental groups after the intervention, although the average score of the experimental group was slightly higher. Self-efficacy scores improved in the post-learning survey compared to the pre-learning survey in both the control and experimental groups; however, the difference between the groups was not statistically significant. Since self-efficacy is commonly reported to be related to online education [29], making direct comparisons between before and after learning is difficult. However, when cardiopulmonary resuscitation and defibrillation e-learning were the outcome factors, self-efficacy improved, which differs from the results of this study [30]. However, in a metaverse-based simulation study of psychiatric nursing, there was no difference in self-efficacy [1], similar to the results of the present study. These findings align with prior literature indicating that self-efficacy gains in virtual environments often depend on the fidelity of the simulation and the degree of active learner engagement [31]. Inconsistencies across studies suggest the need to consider mediating factors such as learner experience level, immersion quality, and task relevance. Since the implementation of metaverse in education is still in its initial stages, owing to the non-implementation of AI-based adaptive learning systems and IoT for immersive virtual-real space interaction [32], it is believed that self-efficacy improved, although there was no significant difference.

There was also no significant difference in self-regulated learning between the control and experimental groups after the intervention, although the experimental group showed a slightly higher average value. Self-regulated learning showed no significant difference between pre- and post-intervention in the control group, but showed a significant difference in the experimental group. In particular, there was a difference in self-regulated learning pre- versus post-intervention among the students. This finding supports studies [33,34] that argue that repeated autonomous practice in the metaverse OR can promote cooperative and independent learning among students, thereby promoting student-centered education.

There was no significant difference in patient safety nursing in the OR between the control and experimental groups after the intervention; however, the pre- and post-intervention differences in both groups were statistically significant. This aligns with Kim and Jeong, who emphasized that structured patient safety protocols can be effectively delivered through both in-person and virtual modalities, suggesting that metaverse-based approaches may be sufficient for foundational safety training [13]. In other words, the educational effect of patient safety nursing in the OR improved in both actual OR clinical practice and metaverse-based OR practice, and the educational effect was similar. It is difficult to make a direct comparison between these teaching modes because very few studies have compared actual clinical practice with metaverse-based practice. However, several studies have confirmed the usefulness of metaverse-based learning [19,35]. Examples include a study that developed a metaverse reality-based family-centered handoff education program for nursing students [36], a study on metaverse hospitalization management for nursing students [37], a metaverse-based simulation education program on schizophrenia nursing [1], and a study that aimed to enhance academic achievement through metaverse-based learning [38]. In a meta-analysis study, e-learning had a very strong and positive effect on the knowledge and skills of participants, but the magnitude and direction of the effect on learning performance differed greatly depending on the method of instruction and situation [39]. Therefore, further diverse and continuous studies are required to evaluate the effectiveness of metaverse-based education.

A notable finding of the present study is that metaverse and clinical practice education showed similar effects. Although all nursing students should experience clinical practice in an actual OR and receive high-quality nursing education, the number of ORs in university hospitals in Korea is limited and the population of nursing students is large, making it difficult for all nursing students to experience OR practice in reality. Therefore, metaverse-based ORs should be used to simulate actual clinical practice in educational programs and achieve the learning outcomes of the course. In addition, metaverse-based educational programs should be developed and applied to various clinical practice courses that include departments where clinical practice is difficult, such as delivery rooms and neonatal units. Future research should further explore how various instructional designs, learner profiles, and clinical domains influence the effectiveness of metaverse-based nursing education. Comparative studies involving other immersive technologies, such as VR/AR and AI-integrated platforms, would also enhance the integrative understanding of digital transformation in clinical training [2]. Implementing the metaverse in the field of education entails various challenges for all stakeholders, including policymakers, instructors, and students. It is important to consider how to approach education to integrate traditional educational and social values to meet the needs of today’s students.

## Limitations

The limitations of this study are as follows. First, because the experimental and control groups were recruited conveniently, exogenous variables could not be effectively controlled. It is possible that students who were active in learning participated in the program. Second, the dropout rate in the control and experimental groups was high, making the study sample insufficient for statistical analysis. Third, since proficiency in patient safety nursing was measured using a questionnaire, caution should be exercised when interpreting the results. Fourth, the development of the metaverse-based OR training program was financially constrained. The total development cost was approximately 50 million KRW (≈ 37,000 USD), which required the prioritization of essential educational components over additional immersive features. As a result, some advanced interactive elements that could further enhance the learning experience were not incorporated.

## Conclusions

This study confirmed that metaverse-based education on OR patient safety nursing, developed using the ASSURE instructional design model, can achieve learning outcomes comparable to those of actual clinical practice. The experimental group demonstrated significant improvements in self-regulated learning and patient safety nursing competencies, while both groups showed gains in self-efficacy and learning transfer motivation. Although no statistically significant differences were observed between the control and experimental groups across most variables, the metaverse-based group consistently reported slightly higher average scores.

These findings indicate that metaverse-based education can be a viable and effective alternative, particularly in settings where clinical placement opportunities are limited. The integration of interactive and autonomous virtual environments helped promote student-centered learning and supported knowledge application in simulated clinical situations. Additionally, the comparable effect of the metaverse-based program to actual clinical practice underscores its potential to supplement traditional methods in nursing education, especially in high-risk or low-access units such as operating rooms, delivery rooms, and neonatal units.

Future studies should expand upon this research by incorporating larger sample sizes, improving technological fidelity through AI and IoT integration, and applying the metaverse to other clinical domains. Such advancements will help refine digital instructional models and strengthen the evidence base for immersive learning in nursing education.
